# SE-ResAutoNet: an attention and residual learning framework for reliable gait-based suspect identification

**DOI:** 10.3389/frai.2026.1838460

**Published:** 2026-08-24

**Authors:** Vaishnavi Munusamy, Sudha Senthilkumar

**Affiliations:** School of Computer Science and Engineering, Vellore Institute of Technology, Vellore, Tamil Nadu, India

**Keywords:** biometric identification, deep neural networks, human gait recognition, person identification, Squeeze-and-Excitation (SE) blocks, suspects/criminal identification

## Abstract

Gait recognition relies on the distinctive walking pattern of the individuals that helps to identify the person accurately even in the long distance or in varied environments without any user interaction. Unlike existing techniques, the method works independently, it does not depend on a person engaging or interacting directly with the system. The SE-ResAutoNet (Squeeze-and-Excitation Residual Autoencoder Network) proposed in this study, an autoencoder framework which adopts SE blocks and Residual Blocks in their architecture for better feature representation and classification accuracy. The attention based - residual learning helps to capture the complex pattern effectively. The SE-ResAutoNet model has achieved an average accuracy of 95.75% across 5-fold cross-validation under normal, slow, and fast walking conditions, along with high precision, recall, and Rank-1 identification performance, demonstrating the consistency and robustness of the model. Furthermore, the proposed framework achieved benchmark accuracies of 97.1% on CASIA-B and 96.32% on OU-MVLP datasets, indicating strong cross-dataset generalization capability. The model also demonstrated computational efficiency with a minimal inference time of 11 ms per frame (77 FPS) and a compact computational cost of 1.5 GFLOPs, highlighting its suitability for real-world video-based surveillance applications. In addition, inclusion of Integrated Gradients highlights the most influential gait regions in the frame. The finding suggests that SE-ResAutoNet achieves an effective balance between accuracy, computational efficiency, and explainability, highlighting its strong potential for deployment in practical biometric and surveillance applications, subject to further validation on larger and more diverse datasets.

## 1 Introduction

Gait analysis, which is a study of how individuals walk, is utilized to detect individuals built on their unique patterns of movement. This technique can be employed in various contexts such as identifying individuals at crime scenes, verifying alibis, tracking persons of interest, and comparing the effects of drugs or alcohol on gait. Both qualitative and quantitative analyses can be performed depending on the available video material, and the angle of view is crucial for accurate comparison. Gait analysis has proven effective even when perpetrators attempt to disguise their walks, which has led to guilt admissions, saving time and costs in investigations and court proceedings.

Gait analysis has a long history in legal proceedings, beginning in 1839 with the first known use in a London court (Jackson, 1839). Over the years, notable developments include Alphonse Bertillon's signaletics in the late 1800s (Justia US Law, 1909), the US Department of Defense HumanID program in the early 1990s, and various landmark cases such as the 1908 Texas robbery (Doyle, 1912) and the 1909 New York extradition of Oscar Slater, where Helen Lambie recognized Slater by his distinctive walk (Wilder and Wentworth, 1918). The methods were expanded in 1918 by Wilder and Wentworth (Smith, 1959), and in 1937, Sir Sydney Smith gave testimony in Scotland, which further advanced the field (Justia US Law, 1971). From the 1971 James Leroy Iverson case (Justia US Law, 1961) to the 1976 conviction of Jerry Mark in Iowa (Justia US Law, 1996), where podiatrist Terry K. Lichty compared the gait of the suspects to shoeprints found at the crime scene, gait analysis has evolved into a crucial forensic tool.

In the 2000s, it gained legal recognition in the UK, Denmark, and Canada, leading to several convictions (Buncombe, 2000; The Argus, 2000; Lynnerup and Vedel, 2005; BCSC, 2008). South Korea adopted this technology in 2014, with significant investments to further develop it (Encode Justice, 2021). The UK established guidelines in 2019, and in 2021, gait analyses contributed to the conviction and sentencing of Mohan Chauhan for the rape and murder of a woman in Saki Naka, Mumbai (Live Law, 2022). This case highlights the effectiveness of gait analysis in linking suspects to crime scenes, thus providing compelling evidence that contributes to successful prosecution.

Technological advancements have further enhanced gait analysis by incorporating machine learning into computer vision systems to improve the accuracy and efficiency. Automated systems can quickly process large volumes of video data and provide real-time analyses and alerts during surveillance operations. Gait recognition systems are increasingly being used in security and law enforcement, airports, and public transportation hubs to detect suspicious activities and identify individuals on watchlists.

Furthermore, academic research continues to expand the capabilities of gait analysis and investigate the integration of deep learning algorithms to refine classification and feature extraction processes. The development of gait-based authentication systems, which provide a constant and subtle way to verify identity, is an additional outcome of the increased interest in biometric authentication technologies. Gait analysis is expected to become increasingly important in security and forensic applications as technology develops, utilizing artificial intelligence (AI) and frontline sensor technologies to expand its applicability and dependability.

Despite these advancements, challenges persist regarding their practical deployment. Gait recognition systems must cope with occlusions, varying camera angles, changes in clothing, and low-resolution footage factors that commonly occur in real-world suspect identification scenarios. Several recent methods, such as GaitSet (Chao et al., 2019) and GaitPart (Fan et al., 2020), model temporal and spatial information from silhouette sequences but struggle with performance degradation under occlusion or extreme viewpoint variations. Other CNN-based approaches lack attention mechanisms and are limited in their capability to crystallize vital informative gait features. These limitations highlight the need for models that are not only accurate but also robust and generalizable under practical surveillance conditions.

To address these challenges, this study introduces a novel deep learning architecture called SE-ResAutoNet (Squeeze-and-Excitation Residual Autoencoder Network), designed specifically for suspect identification using gait. By integrating SE attention blocks and residual learning within an autoencoder framework, the model captures fine-grained, discriminative gait features despite the presence of visual noise or occlusion. This architecture enhances the feature representation and classification accuracy while remaining adaptable to real-time video input.

Despite the promising performance achieved by existing gait recognition approaches, several limitations still remain. Many conventional CNN, transformer, and autoencoder-based frameworks are highly sensitive to viewpoint variation, occlusion, illumination changes, clothing appearance, and walking-speed variations, which affect robustness in real-world surveillance environments. In addition, several deep architectures primarily focus on improving recognition accuracy while introducing higher computational complexity, increased memory consumption, and reduced suitability for real-time deployment. Furthermore, limited attention has been given to jointly integrating discriminative feature recalibration, latent gait representation learning, computational efficiency, and explainability within a unified gait recognition framework.

Squeeze-and-Excitation (SE) attention mechanisms, residual learning, and autoencoder architectures have individually demonstrated strong performance in deep learning applications; however, their unified integration for lightweight and explainable gait-based suspect identification under surveillance-oriented conditions remains relatively underexplored. To address these limitations, the proposed SE-ResAutoNet framework integrates SE attention modules with residual autoencoder learning to enhance discriminative gait representation, preserve fine-grained gait characteristics, and improve robustness against variations in walking speed, illumination, occlusion, and viewpoint changes. Furthermore, the framework incorporates explainability analysis through Integrated Gradients and is designed as a lightweight architecture suitable for real-time surveillance and forensic applications.

While Squeeze-and-Excitation attention mechanisms, residual learning, and autoencoder architectures have individually been explored in deep learning applications, existing gait recognition frameworks rarely combine channel-wise attention-based feature recalibration, residual feature propagation, latent identity-covariate feature decomposition, explainability analysis, and lightweight deployment characteristics within a single unified architecture. The proposed SE-ResAutoNet addresses this gap by jointly integrating these components to improve discriminative gait representation learning, robustness against gait variations, computational efficiency, and model interpretability for surveillance-oriented suspect identification. This paper aims to achieve the following objectives:

- A novel SE-ResAutoNet architecture is proposed by integrating Squeeze-and-Excitation (SE) attention modules with residual learning inside an autoencoder framework to enhance discriminative gait feature extraction for suspect identification.
- The proposed framework incorporates channel-wise attention-based feature recalibration, residual feature propagation, and dual latent feature decomposition to effectively preserve identity-related gait characteristics while reducing the influence of covariate variations such as walking speed, appearance changes, and occlusions.
- A comprehensive pre-processing and data preparation pipeline involving silhouette extraction, temporal sequence standardization, normalization, augmentation, and structured metadata handling is developed to improve robustness and consistency under varying surveillance conditions.
- An explainable and computationally efficient gait recognition framework is designed by integrating Integrated Gradients-based visualization for model interpretability along with a lightweight architecture suitable for real-time surveillance and forensic applications.

In this study, we focused on identifying suspects based on gait technology, using a real-time dataset. Section 2 surveys previous research related to our work. Section 3 delves into the architectural details of the model, followed by the experimental results and analysis, which highlight the experimental outcomes and their performances. The paper concludes with findings and recommendations for future work.

## 2 Related work

### 2.1 Overview of recent gait recognition approaches

Several techniques have been emerged in recent years for gait recognition, these architecture handles different such as noisy background, occlusions, cloth variations and different viewpoints. To obtain discriminative spatiotemporal features from the gait sequences, these models apply hybrid architectures, recurrent units, attention mechanisms, and pose-based encoding.

### 2.2 Attention-based and covariate-controlled gait models

The GA-ICDNet model, which is called “Gate Controlled and Shared Attention ICDNet,” does not perform very good in the aspect of lighting and background variations but uniquely through its modules significantly it improves the gait recognition accuracy. The shared attention module and the use of the covariate feature control gate help in the matter of excessive disentanglement by providing a clearer representation based on spatial and channel information (Chai et al., 2021). The use of a very small memory buffer for past samples has solved the problem of catastrophic forgetting by the iLGaCo model, and also it has made possible the gradual learning of covariate components for identification through gait. It has only considered two factors of covariates, i.e., walking conditions and viewpoints, and is reliant on a small number of historical samples but still it performs quite well with very low memory and computing load (Mu et al., 2020). Whereas, the MSTFE (“Multi-Temporal-Scale Feature Extractor”) model is able to take advantages of slowly and rapidly changing gait information movements to enhance carrying performance in difficult situations. This is useful for obtaining the gait data over different temporal scales. On the other hand, it does not pay much attention to spatial details, on which the robustness against changes in clothes, the objects carried, and the viewing angles might depend (Lin et al., 2020).

### 2.3 Spatiotemporal and CNN-based approaches

Spatiotemporal models that were designed by Su et al. (2021) and Liu et al. (2021), for instance, have the ability to significantly refine spatial features so that the unique characteristics of the subject can be recognized and the changes in gait, clothing, and viewpoints can be easily accommodated. However, these models can face difficulties with changes in clothing and perspectives, which can alter the gait appearance and movement patterns. SelfGait, a dedicated spatiotemporal model, extracts large, varied, and unlabeled gait datasets for pre-training, improving its spatiotemporal backbone representation, especially for individuals wearing outer garments such as coats or jackets.

### 2.4 Silhouette and GEI-based gait recognition models

Although cross-view gait recognition models frequently suffer from poor silhouette quality, they can capture the shape of the subject using global features and refine the details using local features (Hong et al., 2021). Deep Convolutional Neural Networks (CNNs) have the ability to recognize gait even from very far low-resolution images and performance can be further enhanced by adapting the already trained models to new data. Nevertheless, such models might not take into account all divergences and difficulties, for instance, varying backgrounds, lighting, and dressing (Işık and Ekenel, 2021). Another CNN model has shown that Gait Energy Images (GEI) can effectively acquire spatiotemporal information for detection producing very good results in conditions with changing clothes and different viewing angles. The model was evaluated, particularly on the CASIA-B (Yu et al., 2006) gait dataset. However, its applicability in real-world situations may be limited by its assessment using a single dataset (Aung and Pluempitiwiriyawej, 2020).

### 2.5 Local-global feature extraction and hybrid CNN frameworks

To retain spatial data and improve the distinctiveness of visual characteristics for better gait recognition performance, models that extract both local and global features use comprehensive regional information and global visual signals. Local Temporal Aggregation was also integrated. The models face the following real-world challenges, such as noise, occlusion, cross-view discrepancies, and low-quality inputs (Lin et al., 2021b). In dynamic situations where backgrounds and foregrounds change, the model results of CNN plus Mask R-CNN are notably better in terms of accuracy and being less affected by the errors. They still have small data size as a limitation and might still be susceptible to background and lighting changes (Zhang Y. et al., 2021; Inui et al., 2020).

The GaitNet structure comprising of CNN, LSTM, and Mask R-CNN disentangles the appearance, canonical, and position features to help the recognition process. Thus, it is especially suitable for gait detection at low resolution and over long distances. Yet, these benefits might come at the price of difficulties in handling critical modifications like stance, lighting, and occlusion, which are all very important for precise individual recognition (Zhang et al., 2022). On the other hand, in the same-angle situation, the MFINet model which employs a skeletal figure depiction has over 85% accuracy since it captures the temporal dynamics and takes into account the confounding factors in its decision making. However, it may not be very effective in the case of wearing different clothes and point of view changes which can cause alteration in gait appearance and actions (Cosma and Radoi, 2020).

### 2.6 Graph-based and skeleton-driven gait models

When used in UAVs, Graph Convolutional Network (GCN) models increase the operational range and adaptability, while requiring no subject involvement and operating efficiently with low data quality. Pitch rotations make it difficult to identify gait with UAVs, and carrying objects and changing apparel can deteriorate the data quality (Ding et al., 2022). To enhance the accuracy and robustness, especially in challenging situations, the CNN and ResGCN models integrate skeletal poses obtained from RGB images by representing these poses as graphs that capture the spatial and temporal relationships between the limbs and joints. These models can process sequences of varying lengths and integrate the local and global information. Conversely, their resilience to errors in posture estimation, occlusions, and variations in gait speed, stride length, and style remains to be investigated (Teepe et al., 2021).

Using joint heatmaps, models that integrate CNN, LSTM, and GCN outperformed other skeleton-based representations in set-based and sequence- based gait detection tests. Nonetheless, the performance of these methods is highly dependent on the accuracy of the posture estimation, which is quite difficult to perform under conditions when the body parts of different people overlap while they are in motion (Cai et al., 2021). Moreover, with the advantages of large datasets and good physical interpretability, Fully Connected Network (FCN) models are still unable to completely capture the variations in gait appearance caused by walking speed changing, different types of clothing or objects being carried, thus affecting recognition accuracy (Zhang S. et al., 2021). On top of that, these models require a huge amount of training data, which is usually not the case, for the angular softmax and triplet loss to be ascertained. They also neglected the temporal aspects of gait that could have been useful in cross-view analysis (Han et al., 2022).

### 2.7 Temporal and sequence-based deep architectures

With its Micro-motion Capture Module (MCM), the Temporal Part-based GaitPart model targets short range temporal patterns and enhances the detailed learning of spatial aspects, attaining superior results across several industry standards. However, it might have trouble with low-quality silhouettes (Chai et al., 2021). A greater range of views in the training set improves the recognition accuracy of Hidden Markov Models (HMM), which provides a succinct method of representing human motion using Gait Flow Histogram Images (GFHI). Nonetheless, these models do not consider changes in gait appearance brought on by clothes, objects carried, or speed variations, which could affect their success (Chen et al., 2020).

While the GI-ReID model faces challenges in multi-person and occlusion prone environments as well as in various environmental settings, it is very successful in identifying gait independently of clothing by identifying cloth invariant biometric traits and minimizing computational demands (Jin et al., 2022). Using Bayesian optimization to fine-tune the hyperparameters, the merger of Faster R-CNN with RNN outperformed YOLOv3 and Cascade R-CNN in pedestrian identification. The new gait identification technique, which is applicable for both object carried and not carried, poses difficulties in the differentiation of walking styles which are affected by the characteristics of the object, motion, and viewpoints (Ghosh, 2022).

### 2.8 Advanced hybrid and transformer-enhanced models

Gait identification is further enhanced by a new generation of designs. For identity verification, Siamese BiGRU-dualStack (Progga et al., 2024) analyzed the temporal gait patterns using parallel BiGRU branches. To enable effective motion encoding, AttenGait (Castro et al., 2024) uses spatial and temporal attention modules to replace shadows with optical flows. To make occlusion resistance better, SEGait- ConViT (Hrudya and Poornachandran, 2025) merges CNNs with Vision Transformers using gated positional attention and sparse edge-enhanced GEIs. The VPA and MDTE models (Song et al., 2025) that apply dilated convolutions and view-aware attention improve temporal modeling and raise cross-view consistency. For providing noise-robust recognition, LFHEI (Deng et al., 2025) applies a fuzzy histogram descriptor together with frontal view RGB optical flow. To accurately represent subtle dynamics, RDBA-Net (Junaid et al., 2025) incorporates backflow refinement, hybrid attention techniques, and residual dense blocks. Other methodologies use the combination of center loss, spatial attention, and Bi-LSTMs (Junaid et al., 2024) to enhance the discriminative temporal patterns, while multi-scale features along with deep supervision are combined in SAFLFusionGait (Hu et al., 2024). For the purpose of acquiring more evident identity traits, the SMPLGait (Zheng et al., 2022), ParsingGait (Zheng et al., 2023), and XGait (Zheng et al., 2024) models nonetheless incorporate 2D silhouettes, 3D constructions, and parsing masks with the use of hybrid attention and pooling strategies. Moreover, GaitLLM (Yang et al., 2025) has opened a new way by transforming gait sequences into language-like tokens for semantic level modeling and employing frozen LLMs with few trainable parameters, thus, achieving impressive results.

Recent studies from 2025 to 2026 further demonstrate the growing emphasis on transformer integration, cross-view robustness, and lightweight gait recognition frameworks. Mandlik et al. (2025) proposed an enhanced Inception-ResNet and Vision Transformer-based framework for pose-invariant gait recognition, improving robustness under viewpoint variations and complex walking conditions. Similarly, Zhao et al. (2025) introduced a multi-scale transformer with temporal attention mechanisms to enhance cross-view gait consistency and temporal feature representation. Liu et al. (2025) developed a dual-stream interactive framework with multimodal hierarchical aggregation transformers for improving discriminative gait representation under challenging surveillance environments. Furthermore, Hou et al. (2026) proposed Gait Transformer, an end-to-end transformer backbone architecture capable of capturing long-range temporal dependencies and improving large-scale gait recognition performance. These recent developments indicate that modern gait recognition research is increasingly focusing on transformer-enhanced feature learning, multimodal fusion, and computationally efficient architectures for practical surveillance-oriented applications.

### 2.9 Sensor-based and multi-modal gait recognition

On the other hand, inertial-sensor-based methods, like Pedestrian Dead Reckoning (PDR), have become more popular as a complementary technology to vision-based systems for robust tracking in GPS-denied or low-visibility environments. In their paper, (Klein, 2025) reviewed the literature on PDR techniques and pointed out the importance of activity-assisted learning, hybrid models and end-to-end deep learning frameworks that utilize IMU signals for pedestrian localization and understanding of the motion as central to the evolution of the field of PDR.

Nevertheless, these models are still vulnerable to real-world factors such as lighting, background, clothing, viewpoints, and occlusions, as well as being limited by their performance on low-quality data, conditions, and diverse walking speeds. Thus, there is a high demand for lightweight, attention-residual integrated frameworks that can sustain discriminative feature learning and cross-condition robustness, which in turn motivates the design of the proposed SE-ResAutoNet.

### 2.10 Research gap and novelty analysis

Although significant progress has been achieved in gait recognition through CNN-based, attention-based, graph-based, and transformer-based architectures, several challenges remain. Most existing approaches focus primarily on improving recognition accuracy, while limited attention has been given to the simultaneous integration of discriminative feature learning, lightweight deployment, explainability, and robustness against gait variations within a unified framework. To highlight the research gap addressed in this work, a comparative analysis of representative gait recognition approaches is presented in Table 1.

**Table 1:** Research gap analysis of existing gait recognition methods and the proposed SE-ResAutoNet framework.

Method	Attention-based feature learning	Robust feature propagation	Compact latent representation learning	Explainability support	Lightweight deployment
GaitSet (Chao et al., 2019)	No	No	No	No	Moderate
GaitPart (Fan et al., 2020)	Partial	No	No	No	Moderate
GaitGL (Lin et al., 2021a)	Partial	No	No	No	Moderate
OpenGait (Yu, 2022)	Partial	Partial	No	No	Yes
SkeletonGait++ (Fan et al., 2025)	Partial	Yes	No	No	No
GaitLLM (Yang et al., 2025)	Yes	Yes	No	No	No
**SE-ResAutoNet (proposed)**	**Yes**	**Yes**	**Yes**	**Yes**	**Yes**

As shown in Table 1, existing gait recognition approaches typically emphasize recognition accuracy or temporal feature modeling, whereas only limited attention has been given to combining attention-driven feature enhancement, robust feature propagation, compact latent representation learning, model interpretability, and computational efficiency within a single framework. The proposed SE-ResAutoNet addresses this gap by integrating squeeze-and-excitation attention, residual learning, autoencoder-based representation learning, and Integrated Gradients explainability, while maintaining a lightweight architecture suitable for surveillance-oriented gait recognition applications.

## 3 Materials and methods

Gait recognition plays a significant role across various fields like security surveillance, biometric identification and medical diagnostics. The popular existing models such as GaitSet (Chao et al., 2019), GaitPart (Fan et al., 2020), and GaitGL (Lin et al., 2021a), primarily focus on feature aggregation and sequence-based learning, which overlooking the importance of fine-grained feature recalibration. To address these shortcomings, this study proposes SE-ResAutoNet, a deep architecture that incorporates Squeeze-and-Excitation (SE) blocks with Residual Learning within an autoencoder structure to strengthen gait feature representation.

However, SE and residual block are widely adopted in computer vision-based task, i.e., image classification and object detection but their integration into gait recognition still remains limited. SE-ResAutoNet leverages SE modules to adaptively recalibrate channels, allowing the network to emphasize informative gait features and improving resilience to variations and occlusions.

**Figure 1:**
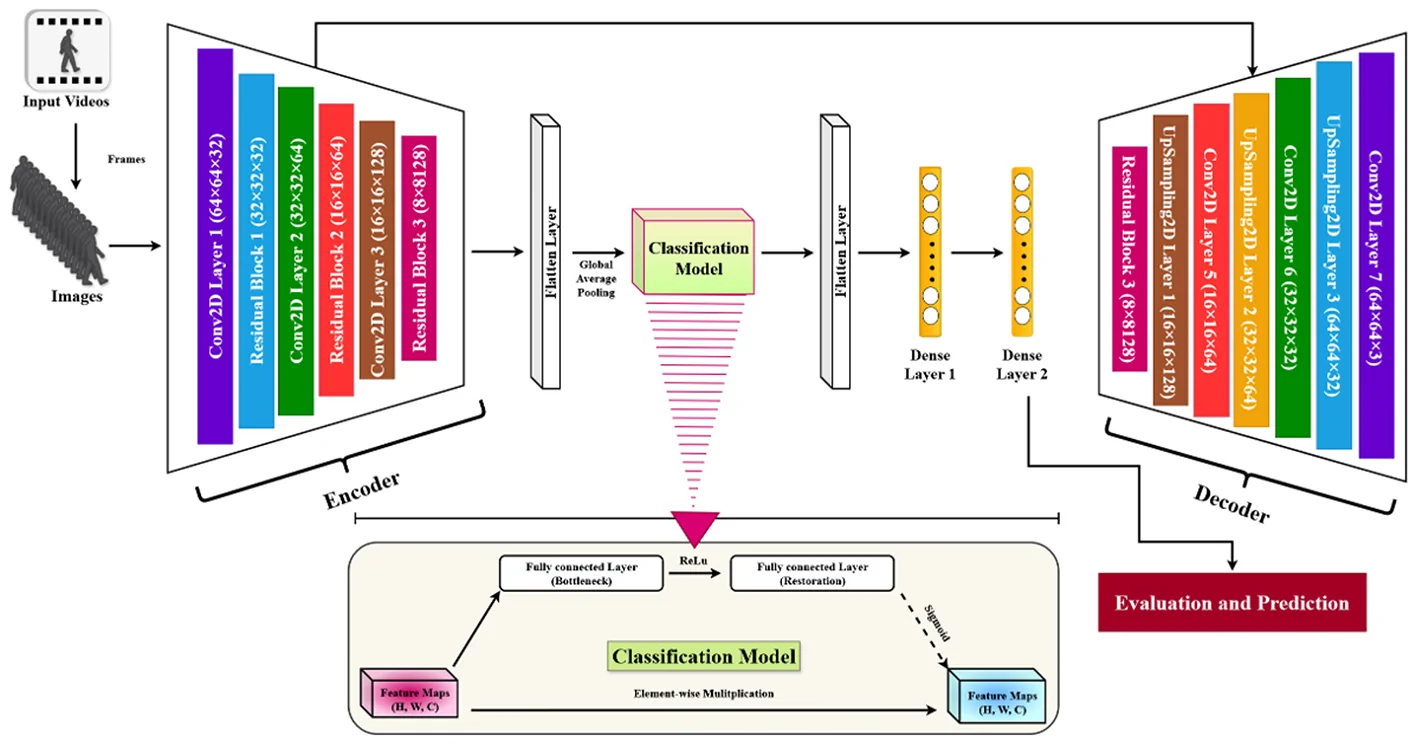
Operational sequence of SE-ResAutoNet model.

For each image, an encoder generates 256 channels latent feature map, further it is split into 192 channels for identity representation and 64 channels for covariate representation, as shown in Figure 1. This decomposition is realized through two parallel 1 × 1 convolutional projection layers, forming identity and covariate subspaces. The identity branch is optimized for the classification via cross-entropy, whereas the covariate branch contributes for reconstruction with MSE. This dual-branch ensuring separation between invariant identity features and dynamic gait variations such as walking speed, cloth variations, and appearance.

Furthermore, the enhanced residual learning strengthens the discriminative learning by supporting deeper gradient propagation and improving representation learning, while SE module addresses dynamic channel-wise feature recalibration and suppress irrelevant noise. Though residual connections slightly increase the computational cost, they meaningfully improve the feature extraction and contributes for the model robustness under varies gait patterns. Euclidean distance has employed to compare the feature embeddings for classification and verification tasks. Dropout layers (0.3–0.5) further applied to limit overfitting and enhance model generalization. Thus, the architecture SE-ResAutoNet balanced well between performance and computational efficiency.

Figure 1 illustrates the complete workflow and architectural design of the proposed SE-ResAutoNet framework for gait-based suspect identification. The framework begins with video acquisition and frame extraction, followed by pre-processing steps including silhouette extraction, resizing, normalization, and augmentation. The processed gait frames are then passed through the SE-ResAutoNet architecture, where residual blocks facilitate deep feature propagation and Squeeze-and-Excitation (SE) attention modules emphasize discriminative gait characteristics. The extracted latent representations are utilized for identity classification and prediction. Finally, Integrated Gradients-based explainability is employed to visualize the most influential gait regions contributing to the model's prediction decisions.

### 3.1 Data pre-processing

The model training was preceded by a meticulous pre-processing operation which aimed at making the input gait data uniform, noise-free, and highly optimized for feature extraction. Each gait video was turned into separate frames at a rate of 25 fps. A silhouette-based background subtraction technique was applied to the dataset in order to eliminate the background scenes and noises. Furthermore, to eliminate artifact, shadow, and contour noise, morphological operations like erosion and dilation were performed, which results in a clean dataset and produces well-defined gait silhouettes.

Frames were resized to 128 × 128 pixels and normalized to [0, 1] range which adds to the numerical stability. To preserve the temporal consistency, each gait sequence was standardized to 30 frames where shorter clips were interpolated and longer ones were uniformly subsampled. This process maintained the temporal rhythm of the gait motion across all the samples.

In addition, to make the model even more robust, the data set was subjected to the augmentation techniques which included, vertical flipping, clipping, and adding noise in the time domain. This guarantees the model to cope with the real-life variations such as different speeds, and different people's faces. A very strict subject-disjoint protocol was used when splitting the dataset, thus ensuring that no subject was represented in more than one subset. The dataset was divided into 70% training, 15% validation, and 15% testing samples.

In summary, the pre-processing stages performed maintained data consistency, created an unbiased dataset which facilitated reliable feature learning and laid a solid foundation for effective feature learning using the SE-ResAutoNet model.

### 3.2 Data preparation

In the data preparation stage, CSV files containing metadata and image annotations were initially processed. These files stored information related to image filenames, subject labels, walking conditions, and sequence details, while the corresponding annotation information was maintained in structured JSON format. The JSON files were parsed to extract the relevant annotation details required for pre-processing and dataset organization. Subsequently, the images were loaded from their respective directories based on the filename references, converted into numerical arrays, and normalized to the range of [0, 1]. The associated labels were transformed into numerical representations using label encoding to prepare the dataset for the model training phase. This pre-processing strategy ensured consistent dataset organization and efficient data handling during training and evaluation.

The pre-processing stage was further performed to standardize the gait frames before feature extraction and classification. Let


$$V=\left\{F_{1},F_{2},\ldots ,F_{n}\right\}$$

represent the input video sequence containing *n* frames. Frame extraction was performed at a fixed interval of every 30th frame and can be represented as


$$F_{s}=\left\{F_{i}|i=30k,k\in \mathbb{N}\right\}$$

where *F*_*s*_ denotes the sampled frame sequence used for further processing.

Each extracted frame was resized to a fixed spatial resolution of 64 × 64 pixels for uniform input representation:


$$I_{r}=\text{Resize}\left(I,64\times 64\right)$$

where *I* represents the original frame and *I*_*r*_ denotes the resized image.

To improve numerical stability during training, pixel intensity normalization was applied as


$$I_{n}=\frac{I_{r}}{255}$$

where *I*_*n*_ represents the normalized image with pixel values scaled to the range [0, 1].

Furthermore, augmentation operations such as rotation, translation, scaling, and noise injection were applied to increase dataset variability and improve model generalization. The augmented image representation can be expressed as


$$I_{a}=T\left(I_{n}\right)$$

where *T*(·) denotes the augmentation transformation function applied to the normalized gait image.

### 3.3 Squeeze-and-Excitation block

An advanced attention approach named squeeze-and-excitation (SE) blocks is based on the understanding of channel interdependencies, which allows adjusting of the channels-wise feature responses mainly directing the focus to the major gait features. The process was done in three steps:

- Squeeze (Global Average Pooling-GAP): For each channel, the average value is calculated reflecting the entire feature map, and that value is replaced with one number that shows the average activation of that channel for the entire grid of values.
- Excitation (Fully Connected Layers): Two dense layers were employed for transmitting the compressed vector. The network determines which features are the most relevant for gait identification through the use of ReLU in the dimensionality reduction of the first layer and the application of sigmoid activation in the creation of the channel-wise significance weights in the second layer.
- Recalibration (Element-wise Multiplication): The process of weighting each channel in the feature map according to its learned weight leads to the amplification of the important gait features along with the suppression of the irrelevant and less relevant ones, thus improving the robustness against occlusions and variations in covariant factors.

In deep learning, SE blocks are popular due to their capability to improve feature representation by analyzing dependencies among channels. Different channels carry different amounts of discriminative information in gait sequences due to factors such as the type of clothing, carrying conditions, and the pace of walking. The SE blocks, by means of feature recalibration, enhance the relevant gait features and simultaneously mask the less important noise. Thus, the output is a stronger feature representation that is independent of the different walking conditions and places.

While SE blocks have been a major part of the architecture in image classification and object detection, their role in gait recognition has not been well-researched yet. Gait recognition is a tricky area with a number of obstacles; these include occlusions, view changes as well as covariates that can cause the gait to appear differently. It is now possible with the integration of SE blocks into SE-ResAutoNet to handle these very problems by polishing the extracted features, thus making the model more robust and better able to generalize over datasets. The integration of SE blocks into SE-ResAutoNet specifically addresses these challenges by refining the extracted features, improving model robustness, and enhancing generalization across datasets.

### 3.4 Residual block

In deep neural networks the skeletal blocks play a vital role by permitting the shortcut connections that skip one or multiple layers. The main merit of them is easing the training of extremely deep networks by vanishing gradient problem alleviation and feature learning efficiency improvement. The architecture contains a residual block, which is composed of two convolutional layers with 3 × 3 filters and “same” padding, then on top are batch normalization and ReLU activation. At last, one more ReLU activation is done when the input block is combined with the output of the second convolutional layer through the shortcut connection. The deeper the network becomes, the more complex features it captures, and the number of filters in the residual blocks is gradually increased from 32 to 128 (32, 64, and 128).

The self-learning technique overcomes degradation in the deep network problem by not just preserving high-level but also low-level features with shortcut connections. In the case of gait recognition, tiny changes in the walking patterns and timing can result in loss of very crucial identity-related information as the features pass through the network. By incorporating residual blocks, SE-ResAutoNet ensures that essential gait features are retained, thereby improving identity consistency across frames.

Besides that, residual connections support the gradient to flow smoothly to the earlier layers during backpropagation thereby preventing the learned features of the deeper layers from getting degraded. This is beneficial for gait recognition, where the fine-grained motion patterns have to be learned over many frames. By leveraging residual blocks, SE-ResAutoNet is able to local and global motion characteristics efficiently which in turn leads to a more robust and discriminative gait representation.

### 3.5 Autoencoder and decoder

In order to accomplish the goal of extracting and reconstructing gait representations that are not only identity-specific but also free from the covariate effects of clothing, carrying conditions, and walking variations, SE-ResAutoNet employs an autoencoder framework operating under the encoder-decoder architecture. The encoder part of the system reduces the input gait images through a series of operations comprising Conv2D layers, max pooling layers, and the application of residual blocks to finally produce a compact latent representation of the key gait characteristics. The convolutional layers play a key role in the process by capturing the complete range of spatial features, both low and high level, thus preserving the information related to the structure of the pattern and the movement, while the residual blocks at resolutions of features (32,32), (16,16), and (8,8) ensure that the very fine-grained patterns of the gait are retained by enhancing feature propagation and preventing the occurrence of vanishing gradients. The max pooling layers, on the other hand, not only reduce the spatial dimensions but also retain the most crucial details, thus leading to the formation of a compact feature space that is distinct for each individual. The structure of the encoder is illustrated in Table 2, which also indicates the contribution of each layer to the processes of compression and feature extraction.

**Table 2:** Detailed layer of encoder and decoder.

Part	Layer	Filters	Kernel/pool size	Strides	Output size
Encoder	Conv2D Layer 1	32	(3, 3)	(1, 1)	(64, 64, 32)
	MaxPooling2D Layer 1	–	(2, 2)	(2, 2)	(32, 32, 32)
	Residual Block 1	32	(3, 3)	(1, 1)	(32, 32, 32)
	Conv2D Layer 2	64	(3, 3)	(1, 1)	(32, 32, 64)
	MaxPooling2D Layer 2	–	(2, 2)	(2, 2)	(16, 16, 64)
	Residual Block 2	64	(3, 3)	(1, 1)	(16, 16, 64)
	Conv2D Layer 3	128	(3, 3)	(1, 1)	(16, 16, 128)
	MaxPooling2D Layer 3	–	(2, 2)	(2, 2)	(8, 8, 128)
	Residual Block 3	128	(3, 3)	(1, 1)	(8, 8, 128)
	Projection Layer	256	(1, 1)	–	(8, 8, 256)
Decoder	Conv2D Layer 4	128	(3, 3)	(1, 1)	(8, 8, 128)
	UpSampling2D Layer 1	–	(2, 2)	–	(16, 16, 128)
	Conv2D Layer 5	64	(3, 3)	(1, 1)	(16, 16, 64)
	UpSampling2D Layer 2	–	(2, 2)	–	(32, 32, 64)
	Conv2D Layer 6	32	(3, 3)	(1, 1)	(32, 32, 32)
	UpSampling2D Layer 3	–	(2, 2)	–	(64,64, 32)
	Conv2D Layer 7	3	(3, 3)	(1, 1)	(64, 64, 3)

The decoder employs Conv2D layers and upsampling techniques to reverse the process of compression from the latent space to reconstruct the gait representation. The gradual 2D upsampling layers enhance the spatial resolution while keeping the identity-related aspects and the following convolution layers consider the quality of the representations in terms of structure and meaning. The last Conv2D layer [3 filters, (3, 3) kernel size] is responsible for the reconstruction by creating an output image of the original resolution. The encoder-decoder configuration contributes to separating the identity-related part of the data from external factors; thus, it increases the robustness of the model in the task of recognition of the gait. Furthermore, residual learning guarantees that the gait features remain intact, while the squeeze-and-excitation (SE) mechanism boosts the feature channels relevant to the task, resulting in the overall process of feature extraction being balanced. This systematic approach strengthens the ability to generalize and ensures strong performance across various datasets and in real-world gait recognition cases.

### 3.6 Classification module

The classification module contains a flatten layer, dense layers, and element-wise multiplication. First, the flatten layer transforms the two-dimensional feature maps into a one-dimensional vector that is compatible with dense layer processing. The dense layers are composed of two fully connected layers: the first one applies a ReLU activation function to prevent bottlenecking issues and the second one employs a softmax activation function to carry out identity classification. Before classification, the feature map of each channel was up-scaled using the channel-wise attention weights produced by the SE block. As a result of this element-wise multiplication, the discriminative gait features are enhanced while the irrelevant ones are suppressed.

### 3.7 Drop out and regularization

To prevent overfitting, dropout layers are applied with a rate of 0.3–0.5 after important dense layers. This technique facilitates the model learning to generalize better, especially when the model is trained on noisy and diverse real-world datasets.

### 3.8 Hyperparameter configuration

The SE-ResAutoNet model was trained using the Adam optimizer with an initial learning rate of 0.001 to achieve stable convergence during training. A batch size of 32 was used, and the model was trained for 50 epochs with early stopping employed to prevent overfitting based on validation loss monitoring. Rectified Linear Unit (ReLU) activation functions were utilized in the hidden layers, while the Softmax activation function was applied in the final classification layer for multi-class prediction. Dropout regularization with rates ranging from 0.3 to 0.5 was incorporated after dense layers to improve generalization performance. The training process was conducted using stratified 5-fold cross-validation to maintain balanced class distribution across the folds. Furthermore, model training and evaluation were implemented using TensorFlow and Keras frameworks on a GPU-enabled computing environment.

### 3.9 Stratified K-fold cross-validation

Estimating the performance of the machine learning model, the dataset was divided into k folds using stratified K-fold cross-validation which guaranteed that the original class distribution was maintained in each part. Each part in this method serves as a validation set while the rest of the parts together form a training set. The performance metrics such as accuracy and loss were computed for every part of the data during the training and evaluation of the model k times. The final performance of the model was derived by taking the average across all segments which contributed a very reliable evaluation. The validation is very useful for classification tasks with imbalanced class distributions.

To ensure consistency and reliability across experimental runs, the same pre-processing pipeline, augmentation strategy, hyperparameter configuration, and training settings were maintained for all 5 folds during cross-validation. The dataset was divided using stratified sampling to preserve balanced class distribution across training and validation subsets. Each fold was independently trained and evaluated while maintaining identical optimization settings, thereby reducing experimental bias and improving reproducibility of the reported performance metrics.

### 3.10 Loss function

The Mean Squared Error (MSE) loss, represented as


$$\begin{array}{c} \text{MSE}=\frac{1}{N}\overset{N}{\underset{i=1}{\sum }}{\left(y_{i}-{ŷ}_{i}\right)}^{2} \end{array}$$ (1)

was used to measure how effectively the autoencoder reconstructed images from their encoded representations. Here, *N* represents the sample count, *y*_*i*_ is the actual value, and ŷ_*i*_ is the corresponding predicted value. By using this particular loss function the autoencoder is being trained to learn how to minimize reconstruction differences which in turn will improve the quality of the features learned by the model.

Conversely, Sparse Categorical Cross Entropy loss, defined as


$$\begin{array}{c} \text{Loss}=-\frac{1}{N}\overset{N}{\underset{i=1}{\sum }}\log\left(\frac{e^{{ŷ}_{i,y_{i}}}}{\sum_{k=1}^{K}e^{{ŷ}_{i,k}}}\right) \end{array}$$ (2)

was utilized for classification tasks involving integer labels, where *K* represents the number of classes. Here, ŷ_*i*_ indicates the predicted probability for the true class *y*_*i*_, and ŷ_*i, k*_ refers to the predicted probability for class *k*. This loss function assists the classification model in aligning the predicted probabilities with the actual labels, thereby enhancing its accuracy in differentiating between classes. Both loss functions are vital for the SE-ResAutoNet architecture: MSE focuses on optimizing feature learning within the autoencoder, whereas Sparse Categorical Cross Entropy improves classification efficacy.

## 4 Experimental result and analysis

### 4.1 Dataset

The dataset used in this study consists of gait video recordings collected from five adult subjects in outdoor environments to simulate real-world surveillance challenges. The recordings were captured under three different walking conditions, namely normal, slow, and fast walking, with variations in illumination and viewing angles. Gait recognition was performed on a frame-wise basis for each video sequence, and extensive data augmentation techniques, including rotation, scaling, translation, brightness and contrast adjustments, and occlusion, were applied to improve model robustness despite the limited number of participants.

The study received ethical clearance from the Vellore Institute of Technology Institutional Ethics Committee (Approval No.: VIT/IECH/2025/16 IECH/15 February 2025/20). All procedures were conducted in accordance with institutional ethical standards and the principles of the Declaration of Helsinki. Written informed consent was obtained from all participants prior to data collection.

Due to privacy and confidentiality considerations associated with biometric data, the raw gait video recordings are not publicly available. Only aggregated and anonymized experimental results are presented in this study, in compliance with institutional data protection policies governing human-subject and biometric research data.

Five subjects recorded the dataset, each under the different conditions of walking and different speeds. The first, second, third, fourth, and fifth subjects gave 16, 19, 21, 14, and 15 video sequences respectively. A single sequence of normal walking (8, 11, 13, 6, and 7 videos, respectively for each subject), slow walking (4 videos for each subject), and fast walking (4 videos for each subject) captures the gait patterns of the subject, where normal walking is the subject's most common speed. From these recordings, a total of 670, 828, 624, 599, and 229 images, respectively for Subjects 1 to 5, were extracted by sampling every 30th frame from each video to reduce redundancy and preserve motion diversity at the same time.

Furthermore, to increase the variability of the data and at the same time to cope with the small number of participants, the data augmentation techniques were very effectively used. The augmentation pipeline was a combination of geometric and photometric transformations such as translation, rotation, scaling, cropping, brightness and contrast adjustments, hue and saturation variations, Gaussian noise, salt-and-pepper noise, blurring, grayscale conversion, zooming, occlusion, and so on. Each of the augmentation methods was performed on 14% of the images, and this resulted in extremely large data volumes. The increased dataset had the following number of images for the different subjects: 4,670 for Subject 1, 4,828 for Subject 2, 5,622 for Subject 3, 3,595 for Subject 4, and 3,226 for Subject 5.

The dataset was split into training and validation in an approximately 80:20 ratio, with a subject-disjoint partitioning for avoiding identity leakage among folds. Specifically, the first, second, third, fourth, and fifth subjects consisted of 3,724 and 946, 3,861 and 967, 4,485 and 1,137, 2,875 and 720, and 2,470 and 756 images for training and validation, respectively. The composition of the complete dataset is given in Table 3.

**Table 3:** Dataset summary and augmentation details for gait recognition model.

Category	Subject 1	Subject 2	Subject 3	Subject 4	Subject 5
Number of videos	16	19	21	14	15
Normal walking	8	11	13	6	7
Slow walking	4	4	4	4	4
Fast walking	4	4	4	4	4
Number of images (converted from videos)	670	828	624	599	229
After augmentation					
Total images	4,670	4,828	5,622	3,595	3,226
Training images	3,724	3,861	4,485	2,875	2,470
Validation images	946	967	1,137	720	756
Augmentation techniques applied (each 14%)					
Rotation, translation, scaling, crop, brightness, contrast, Hue, saturation, Gaussian noise, salt and pepper noise, blurred/low quality images, gray scale, zoom, occlusion					

All the data collection procedures for the custom dataset were performed according to the ethical research standards. The participants were informed and consented which guaranteed voluntary participation, privacy, and secure handling of biometric data through the study.

The SE-ResAutoNet model was trained and validated mainly on this custom dataset, while its comparative evaluation and benchmarking were conducted on the publicly available gait recognition datasets such as CASIA-B (Yu et al., 2006) and OU-ISIR (Takemura et al., 2018). These datasets were only for independent benchmarking, following their respective standard subject-disjoint protocols, and were not merged with the training data. This clear separation upholds full reproducibility and discards any form of cross-subject mixing between splits.

### 4.2 Prediction process

**Figure 2:**
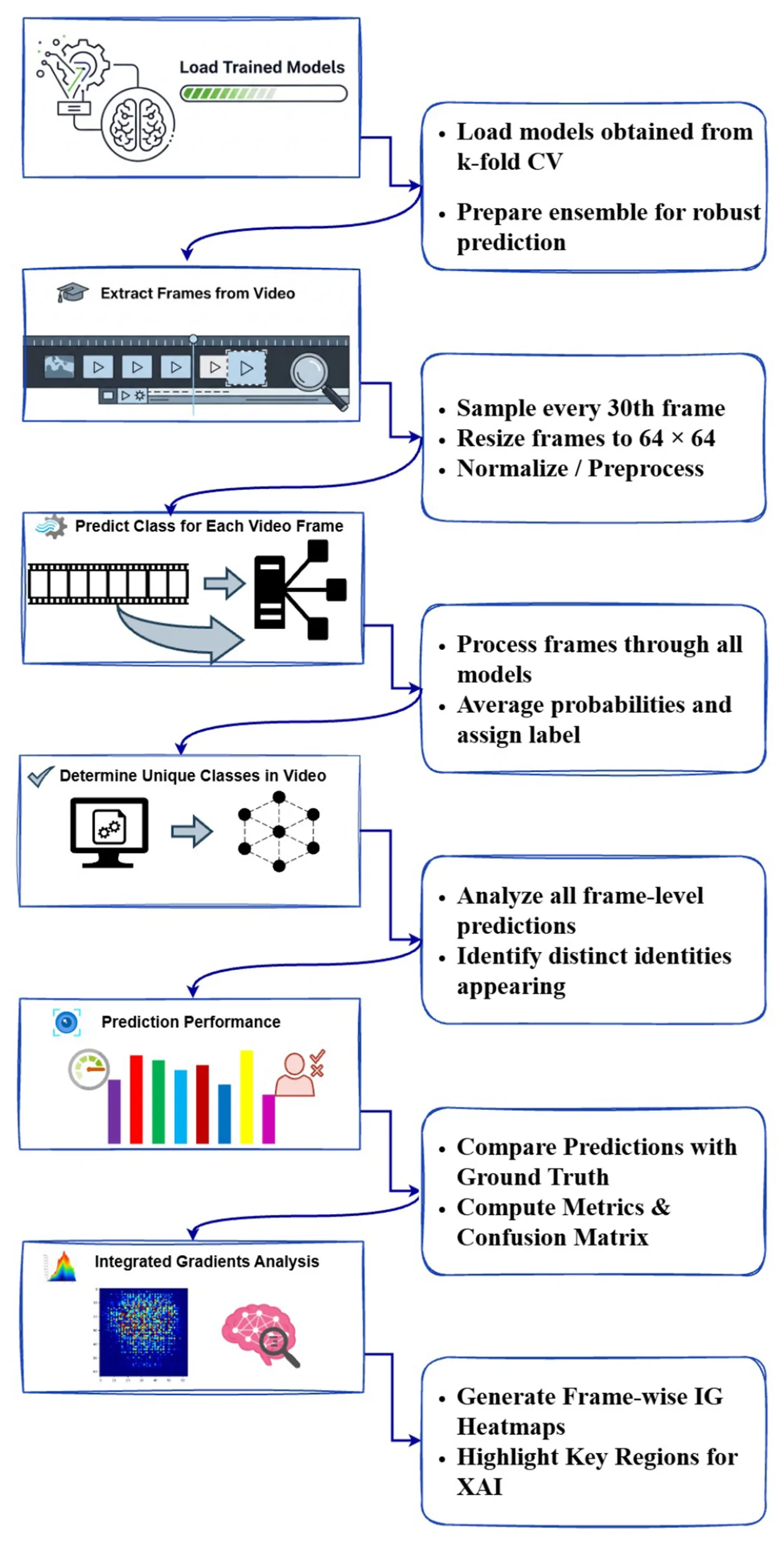
The workflow of the prediction model.

During the prediction, several trained models from cross-validation were used to ensure robustness and performance, strength and reliability. Frames were extracted from the video every 30th frame to manage the data volume and scaled to 64 × 64 pixels to fit the input size of the model. All the models were utilized for each frame, and the results were then averaged to determine the final class for each frame. The video was examined for the unique classes that were indicated by these predictions. The performance of the model was assessed by calculating metrics like precision, accuracy, recall, and F1 score through the comparison of the predicted classes with the actual ground truth labels. A confusion matrix was used to represent the prediction performance visually. Moreover, a heatmap was created to show the areas of each frame that had the greatest impact on the predictions by using Integrated Gradients (Sundararajan et al., 2017), which is an explainable AI-based method. The whole procedure is shown in Figure 2.

### 4.3 Results

#### 4.3.1 Model performance with own dataset

**Figure 3:**
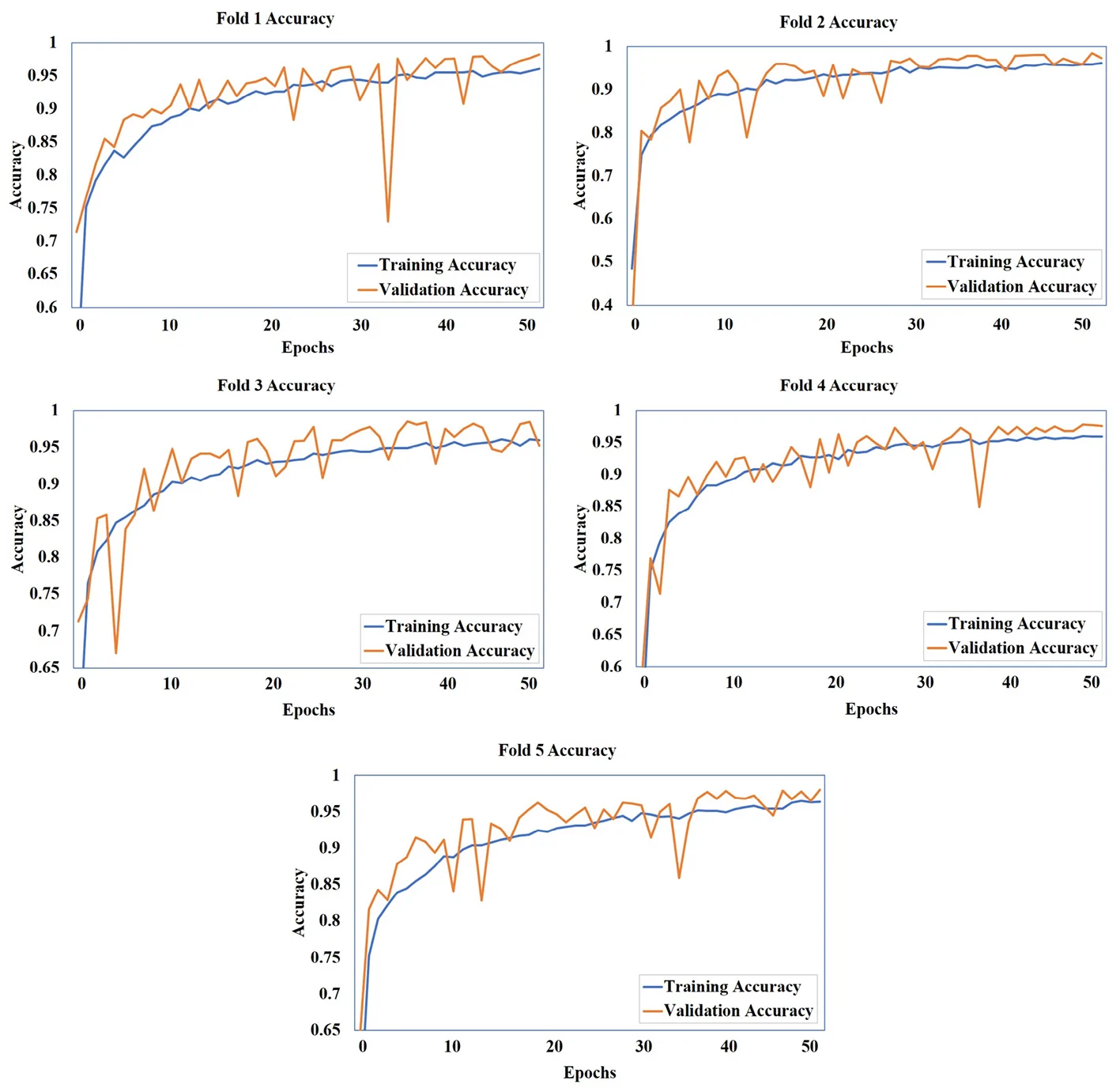
Training and validation accuracy graph across 5 different cross-validation folds.

**Figure 4:**
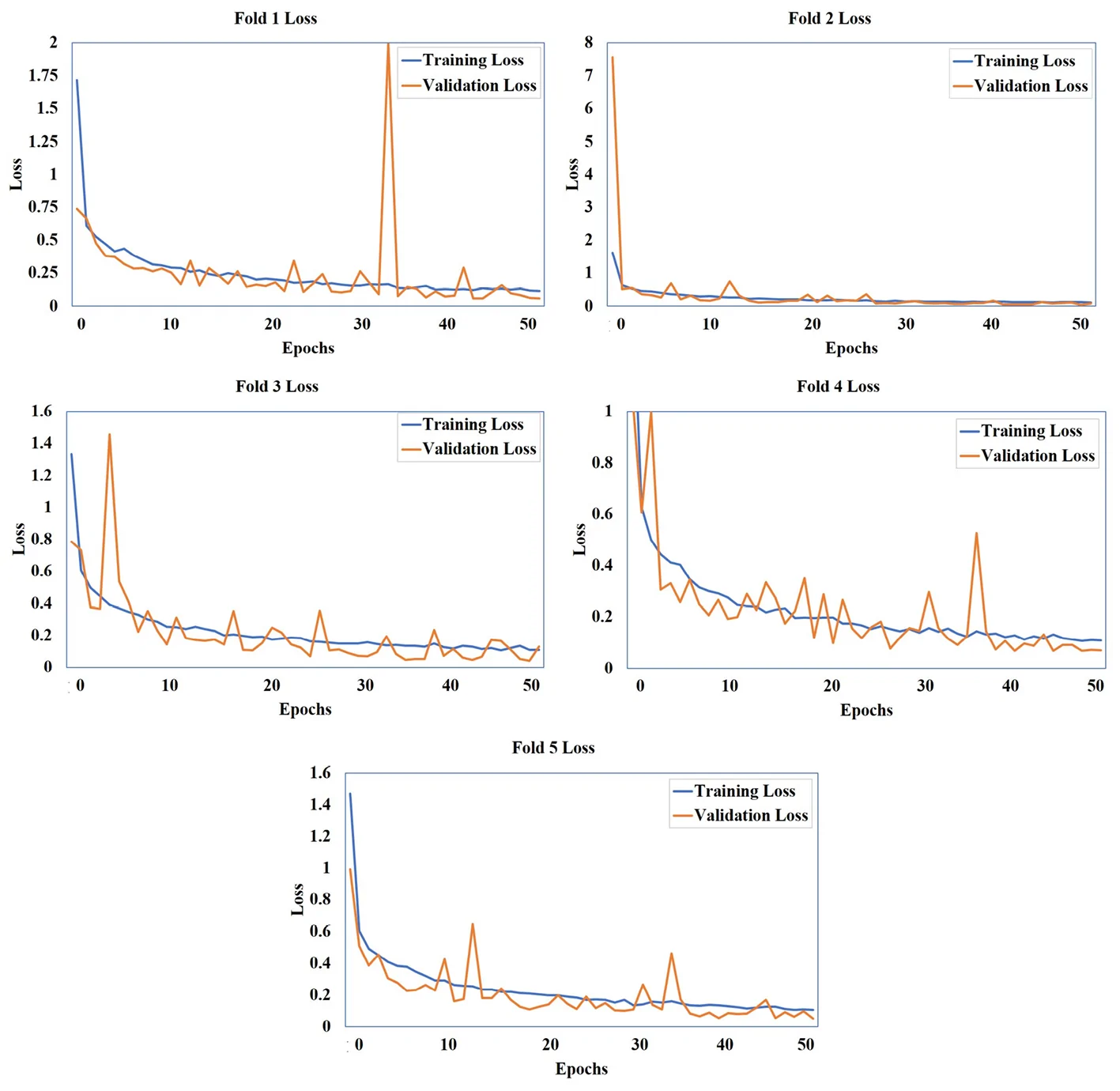
Training and validation loss graph across 5 different cross-validation folds.

**Figure 5:**
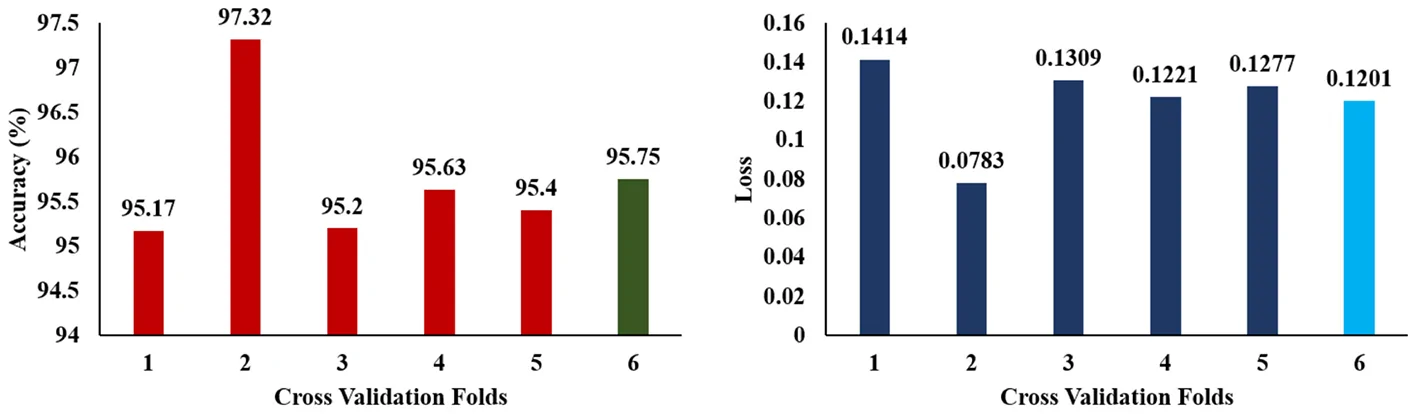
Validation accuracy and loss across each cross-validation fold, including average model performance.

**Figure 6:**
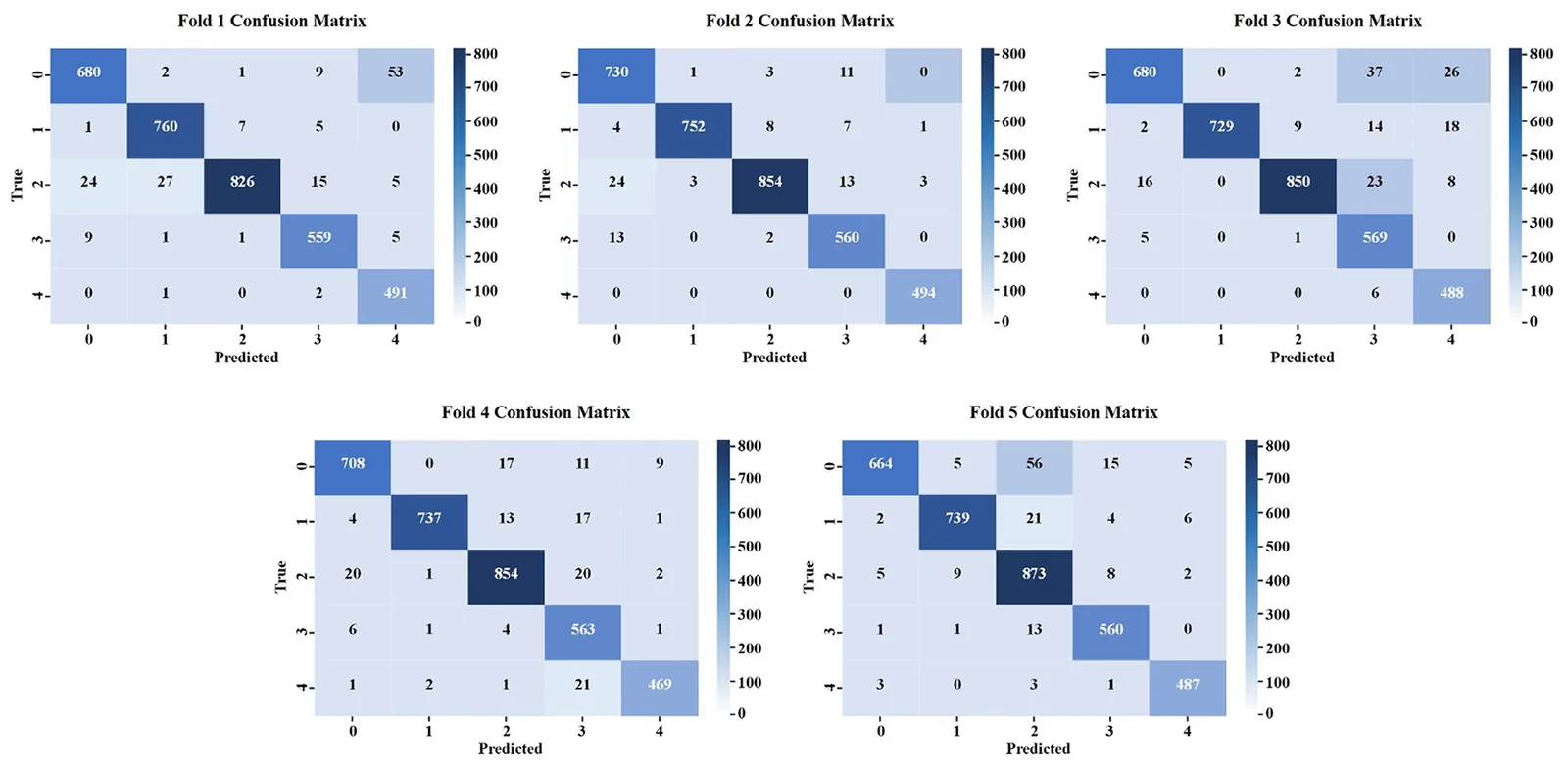
Confusion matrix across five different cross-validation folds.

The performance of the model across the 5-fold cross-validation was as follows: fold 1 achieved an accuracy of 95.17% with a loss of 0.1414; fold 2 achieved an accuracy of 97.32% with a loss of 0.0783; fold 3 obtained an accuracy of 95.2% with a loss of 0.1309; fold 4 had an accuracy of 95.63% with a loss of 0.1221; and fold 5 recorded an accuracy of 95.4% with a loss of 0.1277, as shown in Figures 3, 4. The average accuracy across all folds is 95.75%, as shown in Figure 5, whereas the average loss is 0.1201. This shows a strong model performance with high accuracy and low loss consistently throughout the different folds. For confusion matrix generation, each subject in the dataset was assigned a unique numerical class label during the label encoding stage. Specifically, Subject 1, Subject 2, Subject 3, Subject 4, and Subject 5 were mapped to Class 0, Class 1, Class 2, Class 3, and Class 4, respectively. The rows of the confusion matrix represent the actual ground truth classes, whereas the columns represent the predicted classes generated by the SE-ResAutoNet model. The diagonal elements indicate correctly classified gait identities, while the off-diagonal elements represent misclassifications between different subjects under varying walking conditions. The consolidated confusion matrix of all 5 folds in Figure 6 provides a summary of the classification accuracy and error distribution across each fold.

#### 4.3.2 Robustness of identity recognition across walking conditions

The provided SE-ResAutoNet proposal had to undergo a performance test to confirm that it could be dependable when it came to the recognition of individuals with different walking styles or variations. Three typical walking conditions including Normal, Slow, and Fast, were selected for performance evaluation. This evaluation did not involve the recognition of the type of walking but only the identity established in each condition.

**Figure 7:**
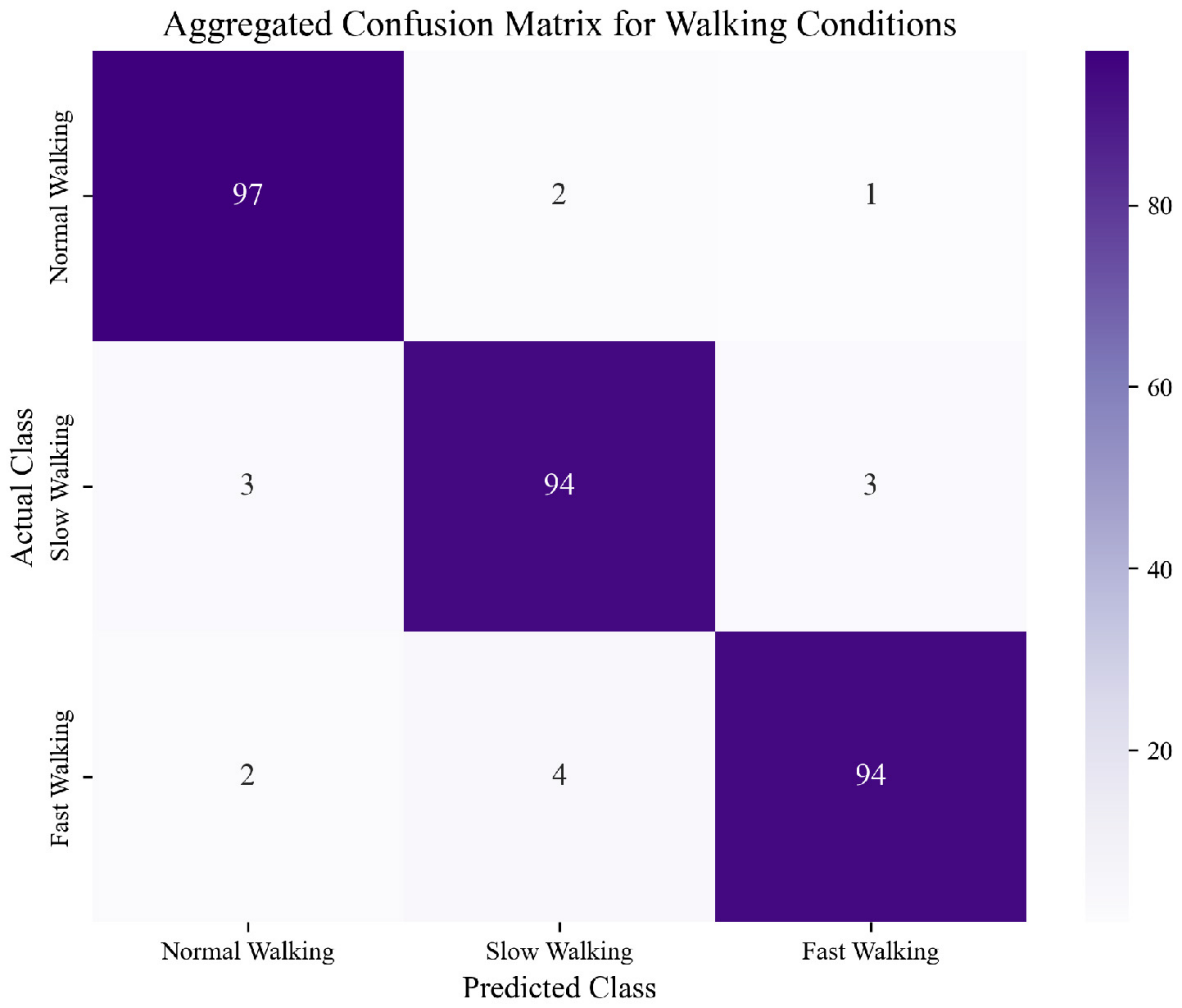
Aggregated confusion matrix illustrating the identity recognition performance of SE-ResAutoNet under different walking conditions (normal, slow, and fast).

The generation of an aggregated confusion matrix (Figure 7) was done through the averaging of the fold-wise identity confusion matrices across three different environmental conditions. The strong diagonal dominance (values exceeding 94% for all conditions) shows that the model is able to consistently identify the same individuals regardless of the walking speed.

The main misidentifications take place between the Slow and the Fast-walking sequences showing up as less distinct in terms of their temporal motion cues. However, the recognition accuracy is still very good the precision and recall being above 94% in all conditions. This is an indication that the SE-ResAutoNet can still keep the identity-discriminative features of the gait even when the gait dynamics vary significantly.

The results of the model presented in Table 4 are impressive and it manages to get an overall accuracy of 95.2% along with the different walking conditions just by using its precision of 94.9%, recall of 95.2%, and the F1-score of 95%. During Normal Walking, the accuracy was very close to 95.1%, with a precision of 94.8%, recall of 95.1%, and F1-score of 94.9%. With Slow Walking the model showed only a slight drop in performance and yielded an accuracy of 93.8%, precision of 93.5%, recall of 93.8%, and an F1-score of 93.6%. Fast Walking scored again a balanced performance in each of the categories by achieving an accuracy of 94%, precision of 93.9%, recall of 94%, and an F1-score of 94%. The results not only highlight the presence of the model as robust in identifying individuals but also as able to do so in a variety of conditions, which is evidenced by the strong generalization and resilience to speed variations.

**Table 4:** Key performance metrics per condition.

Condition	Accuracy (%)	Precision (%)	Recall (%)	F1-score (%)
Normal walking	95.1	94.8	95.1	94.9
Slow walking	93.8	93.5	93.8	93.6
Fast walking	94.0	93.9	94.0	94.0
Overall	95.2	94.9	95.2	95.0

#### 4.3.3 Evaluation metrics

The evaluation of multi-person identification has been carried out to determine the ability of the model in the single frame. Unlike existing single-label classification model, the proposed model supports and assigns multi-label when more than a single person present in a single frame. Table 5 explains the frame-by-frame results, including predicted class, ground truth, correctness of each prediction and its confidence score. The entry “Subject 1, 3” indicates that the frame has two subject (subject 1 and 3) and the model predicted and classified correctly with the confidence score in the range of 0.8–0.9. This outcome demonstrates that the model continuously recognizing the multiple persons in each frame from real-time videos.

**Table 5:** Frame-by-frame prediction accuracy with confidence scores.

Frame number	Predicted class	Ground truth	Correct prediction	Confidence score
1	Subject 1, 3	Subject 1, 3	Yes	0.85
2	Subject 1, 3	Subject 1, 3	Yes	0.87
3	Subject 1, 3	Subject 1, 3	Yes	0.90
4	Subject 1, 3	Subject 1, 3	Yes	0.82
5	Subject 1, 3	Subject 1, 3	Yes	0.80
Overall accuracy/confidence				0.85

#### 4.3.4 Performance comparison of gait recognition models on our dataset

Using our individual dataset, we have made a wide-ranging comparison of the SEResAutoNet model with several others that are considered to be the best in gait recognition so as to determine its performance thoroughly. The results are presented in Table 6, where it is shown that our model has achieved a notable balance between accuracy, model size, and inference speed. In contrast, OpenGait (Yu, 2022) managed to obtain a high level of accuracy of 95.3% along with a moderate number of parameters: 3.78 million and an inference time of 12 ms. Nevertheless, our SE-ResAutoNet not only surpassed but also made it possible for an accuracy of 95.75% to happen while still allowing a parameter count of 5.5 million and having the inference time of 11 ms, which is faster than our parameter count. In the comparison with the exsiting model such as GaitLLM (Yang et al., 2025) (92%, 110M, 22.2 ms) and SkeletonGait++ (Fan et al., 2025) (91.5%, 30M, 14.3 ms), SE-ResAutoNet succeeded in providing highly competitive accuracy even with a significantly lower computational footprint. This combination of recognition performance and lightweight design is what makes it so ideal for real-time and resource-constrained environments, such as surveillance systems, embedded platforms, and mobile applications. SE-ResAutoNet demonstrates promising accuracy and efficiency, indicating its potential suitability for practical gait recognition applications.

**Table 6:** Comparative analysis of gait recognition models with our own dataset.

Model	Accuracy (%)	Parameters (M)	Inference time (ms)
GaitSet (Chao et al., 2019)	94.5	12	11
GaitPart (Fan et al., 2020)	94.9	14	22
GLN (Hou et al., 2020)	95.2	9.46 / 15.62	14
OpenGait (Yu, 2022)	95.3	3.78	12
GaitGL (Lin et al., 2021a)	92.6	18	31
GaitGraph (Chai et al., 2021)	93.2	9	6.7
SMPLGait (Zheng et al., 2022)	85.0	22	16.7
ParsingGait (Zheng et al., 2023)	88.0	25	18.2
XGait (Zheng et al., 2024)	90.2	28	20
SkeletonGait++ (Fan et al., 2025)	91.5	30	14.3
GaitLLM (Yang et al., 2025)	92.0	110	22.2
Multi-scaled deep CNN (Yousef et al., 2024)	93.8	16	19
Disentangled representation learning (Zhang et al., 2019)	94.1	20	24
**SE-ResAutoNet**	**95.75**	**5.5**	**11**

Bold values indicate the results obtained by the proposed system.

#### 4.3.5 Accuracy analysis across benchmark datasets (CASIA-B and OU-MVLP)

In order to further evaluate the generalization capability of SE-ResAutoNet, two publicly available gait recognition datasets, namely CASIA-B (Yu et al., 2006) and OU-MVLP (Takemura et al., 2018), were additionally used for benchmarking. The model was trained exclusively on our in-house dataset and subsequently evaluated on these benchmark datasets without any fine-tuning or domain adaptation. Standard subject-disjoint evaluation protocols recommended for CASIA-B and OU-MVLP were followed during benchmarking. No benchmark samples were incorporated into the training process, thereby preventing data leakage and ensuring an unbiased assessment of cross-dataset generalization capability. As shown in Table 7, several recent state-of-the-art gait recognition models were included for comparative evaluation. On the CASIA-B dataset, BiGRU-dualStack (Progga et al., 2024) and VPA + MDTE (Song et al., 2025) achieved accuracies of 95.7% and 95.06%, respectively, while LFHEI Deng et al. (2025) reported the highest accuracy of 91.9% on the OU-MVLP dataset. In comparison, SE-ResAutoNet achieved accuracies of 97.1% on CASIA-B and 96.32% on OU-MVLP, demonstrating its ability to learn discriminative gait representations that transfer effectively across different datasets. These results indicate that the proposed framework exhibits promising generalization capability and robustness to domain variations while maintaining strong recognition performance on benchmark gait datasets.

**Table 7:** Benchmark comparison of SE-ResAutoNet with state-of-the-art models on CASIA-B and OU-MVLP.

Model	CASIA-B (%)	OU-MVLP (%)
BiGRU-dualStack (Progga et al., 2024)	95.7	87.7
AttenGait-OF (Castro et al., 2024)	91.0	–
VPA + MDTE (Song et al., 2025)	95.06	–
LFHEI (Deng et al., 2025)	90.0	91.9
RDBA-Net (Junaid et al., 2025)	91.6	–
Hybrid Model (Attn + CNN + BiLSTM) (Junaid et al., 2024)	91.03	89.1
SAFLFusionGait (Hu et al., 2024)	92.53	89.8
**SE-ResAutoNet**	**97.1**	**96.32**

Bold values indicate the results obtained by the proposed system.

#### 4.3.6 Ablation study

The ablation analysis further provides insight into the contribution of each architectural component within the proposed SE-ResAutoNet framework. Although standalone supervised architectures such as CNN-only, CNN+LSTM, and CNN+GRU achieved competitive performance, the standalone autoencoder variant demonstrated improved capability in learning compact latent gait representations by preserving discriminative structural gait characteristics while reducing irrelevant background variations. The Residual-only configuration improved feature propagation and training stability, resulting in better performance compared to conventional CNN-based architectures. Similarly, the SE-only model enhanced discriminative feature recalibration through channel-wise attention, thereby improving sensitivity toward informative gait features.

As presented in Table 8, the integration of residual learning and SE attention modules with the autoencoder backbone further enhanced feature discriminability, robustness, and generalization performance. The complete SE-ResAutoNet framework consistently achieved the highest performance across all evaluation metrics, demonstrating that the combined integration of autoencoder-based latent learning, residual feature propagation, and SE attention mechanisms collectively contributes to the overall effectiveness of the proposed architecture rather than increased model complexity alone.

**Table 8:** Multi-metric evaluation of proposed and baseline models.

Model variant	Rank-1 accuracy (%)	Rank-5 accuracy (%)	Precision	Recall	F1-score	AUC-ROC	95% CI (accuracy %)
CNN-only	89.75	96.2	0.88	0.89	0.885	0.915	88.8–90.7
LSTM-only	87.6	94.5	0.86	0.87	0.865	0.901	86.3–89.2
GRU-only	88.3	94.9	0.87	0.88	0.875	0.906	87.0–89.5
CNN + LSTM	90.5	96.7	0.89	0.90	0.895	0.918	89.4–91.6
CNN + GRU	91.1	96.85	0.90	0.91	0.905	0.923	90.0–92.3
Autoencoder	90.2	96.4	0.89	0.90	0.895	0.920	89.0–91.5
SE-only	91.45	97.0	0.89	0.90	0.895	0.926	90.3–92.6
Residual-only	92.87	98.15	0.91	0.92	0.915	0.935	91.6–94.1
**SE-ResAutoNet**	**95.75**	**98.95**	**0.94**	**0.95**	**0.945**	**0.954**	**94.8–96.6**

Bold values indicate the results obtained by the proposed system.

##### 4.3.6.1 Core performance metrics of baseline and proposed models

The performance of the proposed SE-ResAutoNet framework was analyzed using multiple baseline and hybrid architectures to evaluate the contribution of individual network components. CNN-only, LSTM-only, and GRU-only models were considered as baseline architectures, whereas CNN+LSTM and CNN+GRU represented hybrid spatial-temporal learning frameworks. In addition, standalone Autoencoder, SE-only, and Residual-only variants were evaluated to investigate the isolated contribution of latent representation learning, channel-wise attention, and residual feature propagation, respectively. This comparative analysis was performed to determine whether the observed performance improvements originated from the proposed architectural design rather than increased model complexity alone.

Rank-1 accuracy measures the correctness of identification at the first prediction, whereas Rank-5 accuracy indicates whether the correct identity appears within the top five predictions. As presented in Table 8, the proposed SE-ResAutoNet framework achieved the highest Rank-1 accuracy of 95.75% and Rank-5 accuracy of 98.95%, outperforming all baseline and hybrid architectures. These findings demonstrate that the integration of SE attention mechanisms, residual learning, and autoencoder-based latent feature representation significantly improves discriminative gait feature learning, robustness, and generalization under varying gait conditions.

##### 4.3.6.2 Error statistical significance measures

In Table 9, a comparative analysis of different models is provided based on key error and loss metrics which are Loss, Classification Error, Top-5 Error, Log Loss, False Positive Rate (FPR), False Negative Rate (FNR), and overall Misclassification Rate. Traditional models such as CNN-only, LSTM-only, and GRU-only depict the highest errors and losses among all experiments with their classification error between 10.25% and 12.4%. On the contrary, hybrid models (CNN+LSTM, CNN+GRU) offer slight improvements both in classification and top-5 error reduction. An autoencoder usage for feature extraction leads to improvements in both feature compactness and stability but the strongest single models (attention-based and residual) reduce the misclassification the most.

**Table 9:** Quantitative comparison of gait recognition models using error-based metrics.

Model variant	Loss	Classification error (%)	Top-5 error (%)	Log loss	FPR	FNR	Misclassification rate
CNN-only	0.248	10.25	3.8	0.248	0.067	0.060	0.1025
LSTM-only	0.264	12.4	5.5	0.264	0.074	0.070	0.1240
GRU-only	0.258	11.7	5.1	0.258	0.071	0.065	0.1170
CNN + LSTM	0.236	9.5	3.3	0.236	0.063	0.058	0.0950
CNN + GRU	0.229	8.9	3.15	0.229	0.061	0.055	0.0890
Autoencoder	0.241	9.8	3.6	0.241	0.065	0.060	0.0980
SE-only	0.226	8.55	3.0	0.226	0.058	0.053	0.0855
Residual-only	0.208	7.13	1.85	0.208	0.051	0.047	0.0713
SE-ResAutoNet	0.172	4.25	1.05	0.172	0.042	0.050	0.0425

The misclassification rate of Residual-only model is 7.13% while the proposed SE-ResAutoNet goes further down to 4.25% with a total loss of 0.172 proving the synergistic effect of SE attention and residual feature refinement in the framework of autoencoder. The proposed model thus demonstrates the ability to discriminate and minimize errors even in difficult gait variations.

To further validate model consistency and robustness, a 5-fold cross-validation experiment was performed. The fold-wise results with corresponding mean ± standard deviation are summarized in Table 10, demonstrating stable performance across data splits.

**Table 10:** Cross-validation performance metrics with mean ± standard deviation.

Fold	Accuracy (%)	Loss	Precision (%)	Recall (%)	F1-score (%)
Fold 1	95.17	0.1414	94.8	95.1	94.9
Fold 2	97.32	0.0783	95.0	95.3	95.1
Fold 3	95.20	0.1309	94.6	95.0	94.8
Fold 4	95.63	0.1221	95.1	95.4	95.2
Fold 5	95.40	0.1277	95.0	95.2	95.1
**Mean** **±SD**	**95.74** **±0.77**	**0.120** **±0.02**	**94.9** **±0.20**	**95.2** **±0.15**	**95.0** **±0.17**

Bold values indicate the results obtained by the proposed system.

The comparative evaluation reveals that the proposed SE-ResAutoNet model has the advantage of the residual-only baseline in every single fold assessed. The average accuracy obtained by SE-ResAutoNet is 95.74%, as presented in Table 11, which is a big difference compared to the 92.87% of the baseline model. The 95% confidence interval (94.79–96.69%) assures that the performance of the proposed model is very stable and only slightly varies among the different cross-validation folds. Moreover, the enhancement is backed up by a very low p-value (p < 0.001) which is statistically significant and tells us with high confidence that the difference we observed is not likely caused by random variation. Cohen's d = 3.72, which is the effect size, indicates a very large practical impact and implies that adding SE blocks has a considerable influence on the ability of the model to discriminate. Overall, the statistical analysis of SE-ResAutoNet semantics validates it as a reliable and substantial improvement over the baseline architecture.

**Table 11:** Accuracy comparison of SE-ResAutoNet and residual-only baseline with statistical tests.

Metric	SE-ResAutoNet	Residual-only baseline	95% CI	*p*-value	Effect size (Cohen's d)
Accuracy (%)	95.74	92.87	94.79 - 96.69	< 0.001	3.72 (Very Large)

#### 4.3.7 Explainable AI

**Figure 8:**
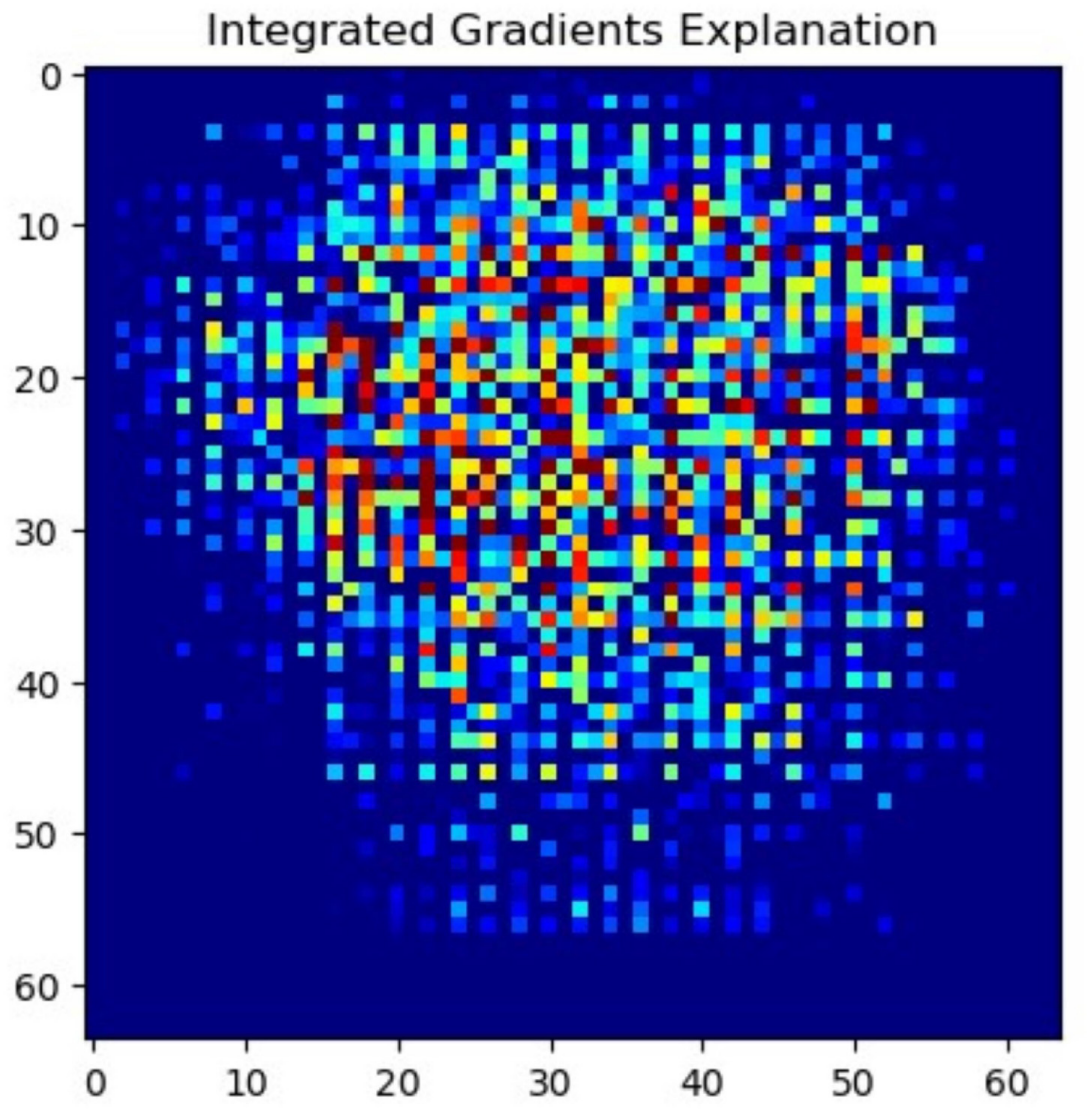
Heatmap visualization of integrated gradients: pixel influence on model decisions.

Explainable AI (XAI) contains various approaches which help to interpret and understand machine learning models better, thus comprehending the rationale of complex models like neural networks that have made their predictions. The XAI relay technique, namely “Integrated Gradients (Sundararajan et al., 2017),” investigates how much each feature has influenced the output by taking output gradients along a path from a baseline to the actual input, thus linking a prediction to the input features. Figure 8 is a heatmap that shows these features for single pixels in a video frame, with hot colors indicating more influential pixels and cold ones depicting less impact. The analysis of the heatmap has led to the discovery of the parts of the frame that had a substantial effect on the decision of the model.

Further analysis of the Integrated Gradients attribution maps revealed that the proposed SE-ResAutoNet framework primarily focused on lower-body motion dynamics, stride patterns, leg movement trajectories, and silhouette boundary regions during gait-based identity prediction. Across different walking conditions, the attribution maps consistently emphasized temporally stable gait regions while suppressing less informative background areas, indicating that the model effectively captured discriminative gait characteristics rather than irrelevant visual artifacts.

In addition to qualitative heatmap visualization, a structured attribution interpretation was performed to analyze the consistency of salient gait regions across different subjects and walking conditions. The analysis demonstrated that regions associated with leg movement and walking posture contributed the highest attribution scores toward prediction decisions, thereby confirming that the proposed framework relies on meaningful biomechanical gait features for classification. This explainability assessment improves transparency and strengthens the reliability of the proposed SE-ResAutoNet framework for surveillance-oriented gait recognition applications.

#### 4.3.8 Computational efficiency

The data presented in Table 12 reveal that the SE-ResAutoNet model not only consumed less computational resources but also made it practically feasible for real-time gait recognition system to be applied. The 11ms per frame and 77 FPS of throughput helped to secure the processing speed that was not at all a trade-off with accuracy. Besides, with 5.5M parameters and 1.5 GFLOPs per frame, the model was able to maintain a good balance between performance and efficiency, while at the same time, having a compact memory footprint of 120MB. The model used 220W of power and 55% of the GPU capacity thus creating space for more workloads. Its latency was composed of 8ms for feature extraction and 5ms for classification ensuring that the decision-making was fast. On the other hand, the support for 256 frames per batch makes it suitable for large applications.

**Table 12:** Computational efficiency and performance metrics of SE-ResAutoNet.

Metric	Value
Inference time	11 ms per frame
Computational complexity	5.5M parameters (1.5 GFLOPs per frame)
Memory footprint	120 MB (FP32 format)
Floating point operations (FLOPs)	1.5 GFLOPs
Throughput (FPS)	77 FPS
Average power consumption	220 W (during inference)
Feature extraction latency	8 ms
Classification latency	5 ms
GPU Utilization	55% (during inference)
Batch processing speed	256 frames per batch
FP16 inference time	9 ms
INT8 inference time	6 ms

In addition, the quantization technique has contributed immensely to the inference speed boost with FP16 still only taking 9 ms and INT8 making it faster at 6 ms per frame. As a result, the SE-ResAutoNet has been optimized to be a durable and efficient model that can provide performance in real-time without needing any extra computational resources.

## 5 Discussion and future works

The SE-ResAutoNet model, an autoencoder architecture integrates both Squeeze-and-Excitation (SE) blocks and residual connections that strengthening the model for better feature representation. The residual connections address the mitigation of vanishing gradient problem, whereas SE module amplifies the important key information in the gait features. This hybrid approach produces more accurate and reliability, also this model yields a potential initiative to use in biometric authentication, security system and surveillance environments.

The proposed SE-ResAutoNet model, achieves accuracy of 95.75% in our own dataset, the model demonstrates strong generalization across various walking patterns and appearance. The consolidated confusion matrix illustrates classification effectiveness. The ablation analysis further confirm that the integration of residual block and SE modules are the key influencer that elevate the performance.

To enhance the transparency, Explainable AI method (Integrated Gradients) were employed to produce heatmaps, which highlights the most influenced gait region and contributes for the decision making. Furthermore, the model also showcases the computational cost, delivering 11 ms per-frame inference (77 FPS), with low memory usage (120 MB, 1.5 GFLOPs). Quantization further accelerates inference processing time reduced to 9 ms (FP16) and 6 ms (INT8).

Compared with computationally intensive deep gait recognition architectures, the proposed SE-ResAutoNet framework maintains a balanced trade-off between recognition accuracy and computational efficiency. Although deeper transformer-based or multi-branch architectures may achieve marginal improvements in recognition performance, such models often require substantially higher computational resources, increased memory consumption, and longer inference times, limiting their applicability in real-time surveillance systems. In contrast, the proposed framework integrates lightweight residual learning and SE attention mechanisms within an efficient autoencoder architecture to improve discriminative gait representation while maintaining low computational complexity and faster inference. The reduced GFLOPs, low memory usage, and accelerated quantized inference further demonstrate the practicality of the proposed model for deployment in resource-constrained biometric and surveillance environments.

Hence, SE-ResAutoNet model, possess a well-balanced between accuracy, efficient and transparency, which make suitable for the real time applications. Although the proposed framework demonstrated promising performance across custom and benchmark gait datasets, large-scale real-world gait variability involving diverse environmental conditions, viewpoints, clothing variations, and subject populations remains a challenging research problem.

Future work will focus on expanding the dataset by incorporating a larger number of subjects under diverse environmental conditions, varying clothing styles, and multiple viewpoints to further enhance the robustness and generalization capability of the proposed framework. Evaluating the model in real-time surveillance systems and integrating privacy-preserving mechanisms will be important steps toward practical deployment in security and surveillance applications while ensuring ethical compliance. In addition, future research will explore advanced hyperparameter optimization strategies and validation on larger, more diverse benchmark datasets to further improve scalability, robustness, and real-world applicability.

## Data Availability

The raw data supporting the conclusions of this article will be made available by the authors, without undue reservation.
